# A time and place for language comprehension: mapping the N400 and the P600 to a minimal cortical network

**DOI:** 10.3389/fnhum.2013.00758

**Published:** 2013-11-12

**Authors:** Harm Brouwer, John C. J. Hoeks

**Affiliations:** Center for Language and Cognition/BCN Neuro-Imaging Center, University of GroningenGroningen, Netherlands

**Keywords:** ERPs, language comprehension, N400, P600, anatomy

## Abstract

We propose a new functional-anatomical mapping of the N400 and the P600 to a minimal cortical network for language comprehension. Our work is an example of a recent research strategy in cognitive neuroscience, where researchers attempt to align data regarding the nature and time-course of cognitive processing (from ERPs) with data on the cortical organization underlying it (from fMRI). The success of this “alignment” approach critically depends on the functional interpretation of relevant ERP components. Models of language processing that have been proposed thus far do not agree on these interpretations, and present a variety of complicated functional architectures. We put forward a very basic functional-anatomical mapping based on the recently developed Retrieval-Integration account of language comprehension (Brouwer et al., 2012). In this mapping, the left posterior part of the Middle Temporal Gyrus (BA 21) serves as an *epicenter* (or *hub*) in a neurocognitive network for the retrieval of word meaning, the ease of which is reflected in N400 amplitude. The left Inferior Frontal Gyrus (BA 44/45/47), in turn, serves a network epicenter for the integration of this retrieved meaning with the word's preceding context, into a mental representation of what is being communicated; these semantic and pragmatic integrative processes are reflected in P600 amplitude. We propose that our mapping describes the core of the language comprehension network, a view that is parsimonious, has broad empirical coverage, and can serve as the starting point for a more focused investigation into the coupling of brain anatomy and electrophysiology.

## 1. Introduction

The aim of the study of language comprehension is to understand how the brain creates meaning from linguistic input. Starting from the lesion-studies of Broca and Wernicke, and subsequent work by Lichtheim and Geschwind, we have learned that the language system is not rooted in a single cortical area, but rather involves a whole network of interconnected regions (a *neurocognitive network*, henceforth). Neuroimaging and lesion studies have since produced a vast collection of data on the cortical organization of language comprehension (for discussions and overviews, see e.g., Cabeza and Nyberg, 2000; Bookheimer, 2002; Price, 2002; Dronkers et al., 2004; Binder et al., 2009; Price, 2010; Andrews, 2011; Turken and Dronkers, 2011). The challenge we now face is twofold: we need to find out what processes are subserved by these areas, and also how these functional processes are ordered temporally.

To arrive at a neurobiological model of language comprehension, a link is needed between time and place of language comprehension in the brain. This means that we will have to find a way to deal with the limitations of the currently available neuroimaging methods; some methods allow for assessing whether cognitive processes are different in kind, and how they evolve over time (e.g., electroencephalography, EEG, and magnetoencephalography, MEG), whereas other methods can be used to pinpoint the location of the areas that are most active during a given cognitive task (e.g., functional magnetic resonance imaging, fMRI, positron emission tomography, PET, and, functional near-infrared spectroscopy, fNIRS). As hemodynamic and electrophysiological measurements are fundamentally different in nature, it is not immediately clear how they should be combined. That is, due to their differences in spatial and temporal resolution, it is often impossible to simply compare them for a given experimental paradigm (cf. Lau et al., 2008).

A more promising strategy is to start with electrophysiology (EEG, MEG), identify and categorize the processes assumed to be reflected in different event-related measurements (Event-Related brain Potentials or ERPs, Event-Related magnetic Fields or ERFs), and then try to find candidate cortical areas or neurocognitive networks that could host them. The success of this “alignment” approach, however, critically depends on the interpretation of ERP/ERF components, and in the literature there is no broad agreement on these interpretations. Take as an example two recent models that have been proposed on the basis of this “process-alignment strategy” (Baggio and Hagoort, 2011; Friederici, 2011). These models disagree on their interpretation of language-related ERP components, and also on where in the brain certain processes are carried out. Baggio and Hagoort (2011), for instance, postulate a complex cortical circuit for word-processing, which they argue is responsible for generating the N400 component (a negative deflection in the ERP waveform which is maximal about 400 ms post-onset). In their model, the N400 is taken to reflect both semantic integration, which they argue takes place in the *frontal* lobe, and the retrieval of word meaning from memory (combined retrieval and integration view), which is carried out in the *temporal* lobe. Baggio and Hagoort make no claims about the processes reflected in the P600 component (a positive deflection in the ERP which is maximal about 600 ms post-onset). Friederici (2011), by contrast, proposes an extensive language comprehension model in which the processes underlying both the N400 component and the P600 component are linked to specific cortical areas. Friederici claims that the N400 component reflects the creation of semantic relations between words or phrases (semantic integration—and critically: no retrieval), and argues that these processes take place in middle and posterior parts of the temporal cortex. The P600 component, in turn, is assumed to reflect the integration of syntactic and semantic information in the Temporo-Parietal Junction or TPJ (see Friederici, 2011, Figure 11).

The interpretation of ERP components and effects thus seems to pose a serious problem, as there is as yet no broad agreement in the language processing literature on what these components mean. This could make the process-alignment strategy a hazardous enterprize, leading to a proliferation of incompatible models. However, we will argue for a research strategy in which one starts from the simplest account of ERP/ERF effects and components, while keeping empirical coverage constant. In a recent paper, Brouwer et al. (2012) present a highly parsimonious account of the two most salient ERP components for language comprehension—the N400 and the P600. They show that their *Retrieval-Integration* account is able to explain a wide spectrum of electrophysiological data on language processing. We will give a brief overview of the Retrieval-Integration account and then apply the process-alignment strategy to derive a *minimal* neurocognitive network of language comprehension that can implement this account. We focus on the *epicenters* (Mesulam, 1990, 1998) or *hubs* (Buckner et al., 2009) of this network that serve to integrate or bind together information from various sub-networks (see also the idea of *convergence zones*, Damasio, 1989). The resulting electrophysiological-anatomic mapping can serve as the starting point for a more elaborate coupling of brain anatomy and electrophysiology. That is, we believe that our proposed mapping forms the core of the comprehension system, which can be extended to account for other language-related ERP components—such as the Early Left Anterior Negativity (ELAN; see Steinhauer and Drury, 2011 for a discussion), the Left Anterior Negativity (LAN; see Kutas et al. 2006), and for instance, sustained negativities like the Nref-effect (van Berkum et al., 2007, see Hoeks and Brouwer, 2014, for a recent account of the Nref as a component)—once we know what processes underlie these components, as well as where these processes are carried out in the brain. Hence, our mapping provides a first step toward such an elaborate neurocognitive model of language comprehension.

## 2. The retrieval-integration account

An ERP is the summation of the post-synaptic potentials of large ensembles (in the order of thousands or millions) of neurons synchronized to an event. When measured from the scalp, continuous ERP signals manifest themselves as voltage fluctuations that can be divided into components. A component is taken to reflect the neural activity underlying a specific computational operation carried out in a given neuroanatomical module (Näätänen and Picton, 1987; Luck, 2005). Components vary in polarity, amplitude, latency, duration, and scalp distribution, suggesting that different components reflect distinct functional processes, carried out in distinct cortical regions. The two most salient ERP components for the study of language comprehension are the N400 and the P600. The N400 component is a negative deflection in the ERP signal that starts around 200–300 ms post-word onset, and peaks at about 400 ms. This component has been taken to index *semantic integration* processes (Brown and Hagoort, 1993; Chwilla et al., 1995; Hagoort and Van Berkum, 2007; Hagoort et al., 2009); words that are semantically incongruent given their preceding context (e.g., *socks* in “He spread his warm bread with *socks*”) produce an increase in N400 amplitude relative to congruent words (e.g., *butter* in “He spread his warm bread with *butter*”; Kutas and Hillyard, 1980), presumably reflecting that they are more difficult to integrate with their prior context. The P600 component, in turn, is a positive deflection in the signal that starts, on average, around 500 ms post-word onset, and reaches its maximum around 600 ms. This component was originally considered to be an index of syntactic reanalysis or repair. Its amplitude has, for instance, been found to increase in response to words that induce a syntactic violation (e.g., *throw* in “The spoilt child *throw* …”) relative to control words (e.g., *throws*; Hagoort et al., 1993). This increased amplitude is taken to reflect the processes involved in repairing the agreement error between the critical verb and its argument. For some time, there appeared to be a clear, one-to-one mapping between the N400 and semantic integration (combinatorial semantic processing), and the P600 and syntactic processing. This mapping forms the core of many neurocognitive models of sentence comprehension (e.g., Friederici, 2002, 2011; Hagoort, 2005; Hagoort et al., 2009, among others). However, in a review of the “Semantic Illusion” phenomenon in sentence processing, Brouwer et al. (2012) have recently shown that an increasing number of experimental findings cannot be explained when adhering to this mapping.

The label “Semantic Illusion” (or “Semantic P600”) is used to refer to a finding in the ERP literature, in which a semantically anomalous sentence does not give rise to an expected increase in N400 amplitude, but rather to one in P600 amplitude (Kolk et al. 2003; Kuperberg et al., 2003; Hoeks et al., 2004). Hoeks et al. (2004), for instance, observed a P600-effect, and no N400-effect, in response to Dutch sentences in which two plausible verb arguments appeared in a semantically anomalous order, as in “De speer heeft de atleten *geworpen*” (lit: The javelin has the athletes *thrown*) relative to “De speer werd door de atleten *geworpen*” (lit: The javelin was by the athletes *thrown*). The absence of an N400-effect is puzzling. The critical verb *thrown* should be more difficult to integrate into the prior context “The javelin has the athletes […]”, as the resulting interpretation of the sentence is in conflict with our knowledge about the world (athletes throw javelins, and not the other way around).

Brouwer et al. (2012) review five models that have been proposed to account for this absence of an N400-effect (taken as absence of semantic integration difficulty), and conclude that none of these models is able to account for the relevant data. They attribute this failure to the assumption that is common to all five models, namely that the N400 component indexes some form of semantic integration or semantic combinatorial processing. Based on recent evidence, Brouwer et al. (2012) argue that the N400 rather reflects a *non-combinatorial* (or *non-compositional*) memory retrieval process (see Kutas and Federmeier, 2000; Federmeier and Laszlo, 2009; van Berkum, 2009; Kutas and Federmeier, 2011, for overviews). On the *memory retrieval view* of the N400 component, N400 amplitude reflects the ease with which the conceptual information associated with a stimulus can be retrieved from long-term memory. In the case of language comprehension, relevant stimuli are typically words, and in this case we refer to memory retrieval as *lexical retrieval*. When dealing with non-linguistic stimuli, however, like an image or a sound, memory retrieval is referred to as *semantic retrieval*. Memory retrieval, lexical retrieval, and semantic retrieval amount to the same thing, and in the remainder of this paper we will use these terms interchangeably to refer to the retrieval of the conceptual knowledge associated with a stimulus (in our case a word) from long-term memory. Ease of retrieval is, among other things, determined by the retrieval cues present in a word's prior context. Retrieval is facilitated if the conceptual knowledge associated with an incoming word is consistent with the conceptual knowledge already activated by the preceding context, and, conversely, retrieval is not facilitated when the features of this word are not activated by the context. For Semantic Illusion sentences such as “De speer heeft de atleten *geworpen*” (lit: The javelin has the athletes *thrown*), the ease with which the lexical features of the critical verb—e.g., *thrown*—can be retrieved from memory depends on conceptual cues in its prior context—e.g., *javelin* and *athletes*—as well as cues from scenario-based world knowledge—e.g., *javelins are usually thrown by athletes*. These retrieval cues should be very similar for the critical verb in the corresponding control sentences, e.g., “De speer werd door de atleten *geworpen*” (lit: The javelin was by the athletes *thrown*). The lexical features of the critical verb—e.g., *thrown*—should thus be equally easy to retrieve in the critical and the control sentences, yielding no difference in N400 amplitude, and hence no N400-effect. This provides a parsimonious explanation for the absence of an N400-effect in Semantic Illusion sentences, but also raises an important question: If the N400 component does not reflect integration—or combinatorial/compositional—processing, then how and when does integration of information from multiple sources (e.g., the meaning of the current word with its prior context) take place? As semantic integration (i.e., the creation of a semantic representation of the language input) is without doubt *the* central task of the language comprehension system, it would be very unlikely that it does not show up in ERPs. Brouwer et al. (2012) hypothesized that these integrative processes are reflected in P600 amplitude. Under this hypothesis, the P600 component is assumed to be a family of (late) positivities that reflect the effort involved in the word-by-word construction, reorganization, or updating of a “mental representation of what is being communicated” (MRC for short). MRC composition requires little effort if the existing representation can be straightforwardly augmented to incorporate the information contributed by the incoming word. It is effortful, on the other hand, when the existing representation needs to be reorganized, supplemented with, for instance, a novel discourse referent, or when the resulting representation does not make sense in light of our knowledge about the world. This last aspect explains the presence of a P600-effect in response to Semantic Illusion sentences like “De speer heeft de atleten *geworpen*” (lit: The javelin has the athletes *thrown*) relative to its control “De speer werd door de atleten *geworpen*” (lit: The javelin was by the athletes *thrown*). Integration of the critical word leads to a representation that does not make sense in light of what we know about the world (javelins are inanimate and cannot throw athletes), and raises the question of what the speaker meant to communicate with this sentence. Did we perhaps misunderstand the speaker, and did the athletes throw the javelin after all? Are we dealing with non-literal language use, as is the case in irony (cf. Regel et al., 2011; Spotorno et al., 2013)? Or did the speaker really mean that some animated javelin was throwing athletes? Hence, in order for the resulting interpretation to be meaningful, we need to recover what the speaker meant to communicate. These recovery processes lead to an increase in P600 amplitude, and hence a P600-effect relative to control. Importantly, our MRC hypothesis of the P600 component predicts that P600 amplitude is sensitive to combinatorial semantic processing in general, and not only to semantic anomaly. This is consistent with evidence from recent studies investigating the incremental processing of atypical, but non-anomalous sentences (e.g., Urbach and Kutas, 2010; Molinaro et al., 2012). These studies report frontally distributed late positive effects for semantically atypical versus typical sentences. Of particular interest are the results of Molinaro et al. (2012), who investigated the processing of different degrees of (a)typicality, and found a significant inverse correlation between P600 amplitude and the “naturality” (the degree to which speakers would produce a given expression) of stimulus sentences, which is clearly consistent with our MRC hypothesis; the less natural an utterance, the more effort it takes to make sense of it, and the higher P600 amplitude.

The views on the N400 and the P600 that were described above are combined in the Retrieval-Integration (RI) account (Brouwer et al., 2012). Under this account, language comprehension proceeds in biphasic N400/P600 cycles, brought about by the retrieval and subsequent integration of the information associated with each incoming word. Every word thus modulates N400 amplitude, reflecting the ease with which its lexical information can be retrieved, as well as P600 amplitude, reflecting the effort involved in integrating a word's meaning with a representation of its prior context. The result of this N400/P600 cycle is an updated representation of what is being communicated in the unfolding discourse thus far, which will itself provide a context for a next word. We expect Retrieval-Integration cycles to be most pronounced for open-class words, as these carry more meaning than closed-class words. However, we also predict closed-class words to modulate N400 and P600 amplitude (see van Petten and Kutas, 1991; King and Kutas, 1995; DeLong et al., 2005; Hoeks and Brouwer, 2014).

## 3. Connecting electrophysiology and anatomy

Retrieval-Integration cycles provide a general and parsimonious account of the elicitation patterns of the N400 and the P600. This sheds light on the *how* and the *when* of comprehension, but not on the *where*. In the second part of this paper, we propose a mapping of the RI account onto a minimal anatomical network. Our approach follows the “process-alignment strategy”; we seek to identify the most parsimonious cortical network that can host the RI-account, while remaining consistent with the extant collection of neuroimaging and lesion studies on language comprehension.

### 3.1. A list of requirements

Based on the assumption that language processing involves continuous Retrieval-Integration cycles, we can specify a list of anatomical “building blocks” that are minimally required to host the RI account in a cortical network. However, before we start with this list, we should define the granularity of the elements to look for. As argued in the introduction, we know that language is not rooted in a single cortical area, but rather in a network of interconnected regions. This is in line with a paradigmatic shift in cognitive neuroscience, in which researchers move from localizationist theories of cognition, toward large-scale, distributed brain networks, called *neurocognitive networks* (see Bressler and Menon, 2010; Meehan and Bressler, 2012, for overviews). The large-scale nature of these neurocognitive networks significantly increases the difficulty of connecting electrophysiology and anatomy. The extent of the language network can, however, still be manageable if we focus on its anatomical and computational *epicenters* (Mesulam, 1990, 1998) or *hubs* (Buckner et al., 2009). These epicenters/hubs are nodes in the network that serve to integrate information from various sub-networks, and are therefore critical gateways for information processing (cf. Buckner et al., 2009). On the basis of such epicenters, we can postulate the following list of requirements in order to create a basic anatomical circuit for language comprehension; we will minimally need two epicenters, and two kinds of pathways connecting them:

1. **An epicenter that mediates *lexical retrieval* (~N400):** This cortical region (or network node) should host or mediate the mapping of word forms to conceptual representations. Conceptual representations are most likely stored in a distributed manner across the association cortices (cf. Pulvermüller, 1999, 2001; Elman, 2004, 2009; Rogers and McClelland, 2004). As such, the full range of activity reflected in N400 amplitude will include the activation of conceptual features in the association cortices, which make up a word's conceptual representation. However, the focus of activity underlying the N400 component is presumed to lie in the *retrieval epicenter*, which serves to “retrieve” and “tie together” these conceptual representations of incoming lexical items, so they become available for later synthesis.
2. **An epicenter that mediates *mental representation composition* (~P600):** This cortical region should host or mediate the integration of the lexical information (retrieved via the retrieval epicenter that was described above) with the existing mental representation of previous input, resulting in a mental representation of what is communicated in the discourse thus far. This specific *integration epicenter* thus hosts or mediates the combinatorial/compositional processes involved in meaning construction, and is assumed to initiate the generation of the P600 component. Again, this area serves as an epicenter, and the full range of activity reflected in P600 amplitude may include activity from other areas as well.
3. **A white matter tract containing fibers that connect region (1) to (2):** This (bottom-up) pathway serves to connect a word's conceptual representation, as retrieved in region (1), to region (2) for integration with a representation of its prior context.
4. **A white matter tract containing fibers that connect region (2) to (1):** This (top-down) pathway connects the newly formed representation that is active via region (2) to (1), thereby providing a context for the retrieval of a next word's meaning. Theoretically, the function of this pathway could be subserved by the white matter tract described in (3) if it were bi-directional, containing fibers that connect (1) to (2), and vice versa.

Provided this list of anatomical requirements, we can try and identify candidate epicenters and white matter tracts. On the basis of several large-scale reviews on the cortical organization of the comprehension system (e.g., Bookheimer, 2002; Friederici, 2002, 2011; Dronkers et al., 2004; Hickok and Poeppel, 2004, 2007; Vigneau et al., 2006; Lau et al., 2008; Shalom and Poeppel, 2008; Turken and Dronkers, 2011), we want to propose two candidate epicenters: the left posterior part of the Middle Temporal Gyrus (lpMTG; BA 21) as *retrieval epicenter* (1), and the left Inferior Frontal Gyrus (lIFG; BA 44/45/47) as *integration epicenter* (2).

### 3.2. Left posterior MTG—an epicenter/hub for lexical retrieval

Evidence from neuroimaging as well as from lesion studies points toward the lpMTG as an epicenter for lexical retrieval (Cabeza and Nyberg, 2000; Bookheimer, 2002; Dronkers et al., 2004; Lau et al., 2008; Binder et al., 2009; Price, 2010; Turken and Dronkers, 2011). Dronkers et al. (2004), for instance, found that aphasics with lesions in the lpMTG suffered from difficulties in word-level comprehension. They argue that this might be the case because the mapping between word-form and conceptual knowledge (=lexical retrieval) is lost in this patient group. Findings from neuroimaging studies are consistent with this hypothesis. Cabeza and Nyberg (2000), for instance, found in a review of functional PET and fMRI studies that both spoken and written word recognition consistently activates the lpMTG, independently of whether words were presented in isolation or as part of a sentence. In addition, Lau et al. (2008) conducted a large-scale review of neuroimaging research on lexical semantic priming and reported that studies consistently found effects in the MTG. More specifically, they concluded that the MTG was the only region showing effects of semantic priming at both short and long (>600 ms) Stimulus Onset Asynchronies (SOAs), which supports the view that the MTG is the generator site of the N400 component. Further evidence for the lpMTG as an epicenter for retrieval comes from MEG studies that investigated the magnetic field equivalent of the N400—the N400m—and used source modeling techniques to localize its generator site. These studies have consistently identified the lpMTG to be involved in the generation of the N400-effect (e.g., Halgren et al., 2002 see Lau et al., 2008, p. 927, for a brief overview). Finally, recent work using connectivity analysis by Turken and Dronkers (2011) provides an important indication for the role of the lpMTG in a network for lexical retrieval. They found that the lpMTG showed a particularly rich connectivity pattern to areas in the frontal, parietal, and temporal cortices of both hemispheres (see Binder et al., 2009; Buckner et al., 2009; Koyama et al., 2010 for similar findings), which is consistent with the idea that the posterior MTG is an epicenter that serves to “retrieve” and “tie together” conceptual knowledge that is represented in a distributed manner across the association cortices.

### 3.3. Left IFG—an epicenter/hub for mental representation composition

It has long been known that the lIFG plays a crucial role in sentence comprehension, but it is still disputed what this role precisely entails (see Grodzinsky and Santi, 2008; Rogalsky and Hickok, 2011, for recent overviews). For instance, Rogalsky and Hickok (2011) point out that a substantial part of the lIFG, Broca's Area (BA44/BA45), has been hypothesized to support syntactic movement (Grodzinsky and Santi, 2008), hierarchical processing and phrase structure building (Friederici, 2009), order-related linearization processes (Bornkessel-Schlesewsky et al., 2009), working memory (Buchsbaum et al., 2005; Buchsbaum and D'Esposito, 2008), cognitive control (Novick et al., 2005) semantic unification (Hagoort, 2005), and thematic role checking and reanalysis (Caplan et al., 2008a,b). Others have stressed the role of the lIFG in the control of memory (Badre and Wagner, 2007). These hypotheses are diverse, and some even appear to be outright incompatible. However, we would like to suggest that it is not necessary to choose between these hypotheses, because the lIFG subserves—to a certain degree—all of the hypothesized processes (see Seghier, 2013, for a similar multi-functional approach toward defining the function of the Angular Gyrus, AG). That is, we propose that the lIFG is host to, or mediator of various types of processing involved in creating and maintaining a mental representation of what is communicated. The word-by-word construction, reorganization, or updating of this mental representation involves the accommodation of new discourse entities, establishing a relation between entities and assigning them a thematic role, revising already established relations, revising already established thematic roles, resolving conflicts between information sources (e.g., with respect to world knowledge), and so on see Brouwer et al., 2012, p. 138). This list subsumes the processes listed by Rogalsky and Hickok (2011), and includes syntactic processes, as well as (working) memory related processes, semantic processes, and control processes. However, our hypothesis is different from the one recently put forward by Bornkessel-Schlesewsky and Schlesewsky (2013), who argue that the lIFG does not sub-serve any linguistic processing at all. On their account, the lIFG only sub-serves cognitive control functions. We find it difficult to reconcile a *cognitive control*-only account of the lIFG with evidence that implies it in combinatorial semantic processing (see the evidence reviewed in Hagoort et al., 2009, for instance).

It is possible that different sub-processes of MRC construction are mediated by or carried out in different, potentially overlapping subparts of the lIFG. Hagoort (2005), for instance, assumes the pars triangularis (BA 45) and the pars orbitalis (BA 47) to be involved in semantic processing, and again BA 45 and the pars opercularis (BA 44) in syntactic processing. Friederici (2009), in turn, suggests that BA 44 supports “simple” hierarchical structure processing, and that the frontal operculum (located between the anterior insula and lateral opercular part of the IFG, cf. Anwander et al., 2007) is involved in more complex local phrase structure building. Elsewhere, Friederici (2011) also argued for such a functional parcellation of the lIFG, and pointed out that debates on the specific role of BA 44 and BA 45 could be resolved on the basis of further subdivision by the type of neurotransmitter receptors found in these areas; BA 44 can be subdivided into a dorsal (BA 44d) and ventral (BA 44v) part, and BA 45 in a more anterior (BA 45a) and more posterior part (BA 45p). Provided this more granular subdivision, conflicting functions allocated to, for instance, BA 44, could actually coexist when one is allocated to BA 44d and the other to BA 44v. Consistent with this idea, recent studies into the organization of the lIFG have revealed a rather complex neuroarchitectural parcellation of the area and its adjacent regions (e.g., Amunts et al., 2010; Amunts and Zilles, 2012). This anatomical parcellation may underlie a fine-grained functional topology within the lIFG. It has been suggested that such a functional topology may be organized in a systematic manner. Hagoort (2005), for instance, suggests that the lIFG is organized in an anterior-ventral to posterior-dorsal gradient, in which semantic processing is subserved in BA 47 and BA 45, syntactic processing in BA 45 and BA 44, and prosodic processing in BA 44 and partially in BA 6. In their two-process model of memory control, Badre and Wagner (2007) also postulate an anterior-ventral to posterior-dorsal gradient underlying lIFG organization, linking BA 47 to controlled access to stored conceptual representations, and BA 45 to domain-general post-retrieval selection processes. Although it remains to be seen if and how well this gradient unifies with the one proposed by Hagoort (2005), for instance through subdivision of the overlapping Brodmann areas, they both underline the idea of a systematic, functional organization of the lIFG.

To arrive at a more fine-grained functional topology of the lIFG, a more focused and systematic investigation of its subdivision is required. Linking the P600 component to the lIFG opens up a new domain of study, where characteristically different types of P600s (in terms of scalp distribution, amplitude, onset, duration, etc.) can be mapped onto different parcels of the lIFG. Such an endeavor will increase both our understanding of the functional topology of the lIFG, as well as our understanding of the different processes underlying the P600 component. A starting point for such an investigation could be the aforementioned anterior-ventral to posterior-dorsal gradient proposed by Hagoort (2005). If this organizational gradient is correct, we would expect more semantically involved MRC processes to lead to increased activity in BA 47 and BA 45, whereas more structurally involved processes should lead to increased activity in BA 45 and BA 44. Moreover, these different types of processing should be apparent in characteristically distinct P600 modulations.

It has proven difficult to localize the neural generators of the P600 component (Friederici, 2011). Attempts at reconstructing these generators using source localization have identified the middle temporal gyrus and the posterior part of the temporal lobe as generator sites for the P600 (Kwon et al., 2005; Service et al., 2007), which is inconsistent with our hypothesis. However, a number of studies using fMRI have linked the P600 to the lIFG (see, van de Meerendonk et al., 2011, for a discussion). Preliminary results from an fMRI study done in our lab are consistent with these findings, and show that the “Semantic Illusion” sentences that produced a P600 effect in the Hoeks et al. (2004) study, also induced increased activity in the pars orbitalis (BA 47) of the lIFG. This correlation between activation in the lIFG and P600 amplitude supports our hypothesis that the P600 is generated in the lIFG. In the discussion, we derive several predictions that can be used to confirm or validate our hypothesis.

### 3.4. Connecting the two epicenters: from retrieval to integration and back

Once the lexical knowledge associated with an incoming word is retrieved by the lpMTG, it needs to be connected to the lIFG for integration. This requires a white matter pathway from the temporal to the frontal lobe. The lIFG then integrates the meaning of the incoming word with a representation of its prior context into a representation of what is being communicated. Importantly, the updated mental representation subsequently serves as a context for the retrieval of the next word's meaning, requiring a white matter pathway back, from the frontal to the temporal lobe.

Traditional anatomical models of language assumed that the major white matter pathway connecting the temporal cortex to the IFG was the *arcuate fasciculus*. Recent tractography studies using Diffusion Tensor Imaging (DTI), however, have led to the identification of a more extensive structural connectivity pattern between these two areas (see Catani et al., 2005; Saur et al., 2008; Makris and Pandya, 2009; Turken and Dronkers, 2011, among others). The general view that emerges from these studies is that the inferior frontal cortex and the temporal cortex are wired together by means of a dorsal and a ventral pathway that can each be subdivided into two sub-pathways. The first dorsal pathway connects the posterior parts of the MTG (and the STG; BA 22) to the pars opercularis (BA 44) via the *arcuate fasciculus* and the *superior longitudinal fasciculus*. The second dorsal pathway connects the posterior STG via the same fiber tracts to the premotor cortex. As for the ventral pathways, one connects the MTG (and the STG) to the pars triangularis (BA 45) via the *extreme fiber capsule system*, and the other connects the anterior part of the STG to the frontal operculum via the *uncinate fasciculus* (the anterior and posterior parts of the temporal lobe are itself connected through the *inferior* and *medial longitudinal fasiculi*). Unfortunately, DTI does not allow us to determine the directionality of white matter pathways (see Friederici, 2011). Nonetheless, three out of the four fiber tracts that we have just described connect the temporal cortex to the inferior frontal cortex (one dorsal pathway connects the STG to the premotor cortex), which provides us with three candidate pathways for our network.

The functional role of these different pathways is, however, still a matter of debate (Hickok and Poeppel, 2004, 2007; Saur et al., 2008; Friederici, 2009, 2011, 2012; Weiller et al., 2009; Baggio and Hagoort, 2011; Tyler et al., 2011; Bornkessel-Schlesewsky and Schlesewsky, 2013). Hickok and Poeppel (2007), for instance, assume in their dual stream model of speech processing that the dorsal pathway maps acoustic speech signals onto articulatory networks in the frontal lobe, whereas the ventral stream sub-serves speech comprehension. Friederici (2012), on the other hand, attributes different functions to the sub-pathways of the dorsal and ventral fiber tracts, suggesting that whereas the dorsal sub-pathway connecting the temporal lobe to the premotor cortex might indeed be involved in mapping acoustic speech signals to articulatory networks (in line with Hickok and Poeppel, 2007; Saur et al., 2008), the dorsal sub-pathway connecting the temporal lobe to the pars opercularis (BA 44) is likely to be involved in the processing of syntactically complex sentences and the delivery of top-down predictions to the temporal lobe. As for the ventral pathways, Friederici assumes the sub-pathway connecting the temporal lobe to the pars triangularis (BA 45) to be involved in the transfer of semantic information from temporal to frontal regions, and the sub-pathway connecting the STG to the frontal operculum in syntactic information transfer. Bornkessel-Schlesewsky and Schlesewsky (2013) have recently challenged the accounts by Hickok and Poeppel (2007) and Friederici (2012), and put forward a new ventral-dorsal framework for comprehension, in which the dorsal stream engages in time-dependent combinatory processing, including syntactic structure building, whereas the ventral stream sub-serves “time-independent identification and unification of conceptual [actor-event (AE)] schemata” (Bornkessel-Schlesewsky and Schlesewsky, 2013, p. 67). This framework is attractive as the authors show that it solves several puzzles regarding the requirement for a dorsal stream in the ventral-dorsal stream literature. However, the role attributed to the ventral stream seems to be a direct instantiation of the “plausibility processing”-stream of the extended Argument Dependency Model (Bornkessel and Schlesewsky, 2006; Bornkessel-Schlesewsky and Schlesewsky, 2008), the existence of which has been challenged in the literature (Stroud, 2009; Stroud and Phillips, 2011; Brouwer et al., 2012). Moreover, a core assumption of this framework is that the role of the lIFG is restricted to cognitive control, which is problematic for reasons discussed above. Hence, the validity of this new framework remains open for close scrutiny.

Given the lack of consensus on the functional role of the dorsal and ventral pathways, it is at present difficult to decide which pathway supports the connection of information retrieved in the lpMTG to the lIFG for integration, and which serves to provide a context for retrieval. On a speculative note, there seems to be at least some agreement on the involvement of the ventral pathway in form to meaning mapping (Hickok and Poeppel, 2007; Friederici, 2012; Bornkessel-Schlesewsky and Schlesewsky, 2013), which supports the idea that this pathway may sub-serve the connection of information retrieved in the lpMTG to the lIFG. As for the dorsal route, Friederici's suggestion that one of the dorsal sub-pathways is, among other things, involved in delivering top-down predictions from the frontal to the temporal lobe, is consistent with the idea that the dorsal route is involved in providing a context for retrieval. However, it remains to be seen how this could be reconciled with the proposals put forward by, for instance, Hickok and Poeppel (2007) and Bornkessel-Schlesewsky and Schlesewsky (2013).

In summary, the specific roles of the ventral and dorsal pathways in Retrieval-Integration cycles are as of yet unclear, but their presence does indicate that there is white matter connectivity between the temporal cortex and the inferior frontal cortex that could implement the required circuitry.

### 3.5. A functional anatomic retrieval-integration cycle

**Figure 1:**
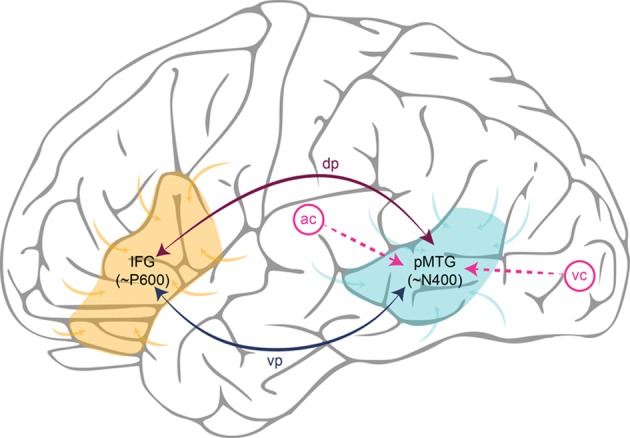
**Schematic illustration of a Retrieval-Integration cycle in the left hemisphere**. Words reach the posterior Middle Temporal Gyrus (pMTG) via the auditory cortex (ac) or the visual cortex (vc), depending on whether the linguistic input is spoken or written. The pMTG retrieves the lexical information associated with a word from the association cortices (generating the N400). The retrieved information is then connected to the Inferior Frontal Gyrus (IFG) via one of the white matter tracts in either the dorsal pathway (dp) or the ventral pathway (vp). The IFG integrates this information with a representation of the prior context into an updated representation of what is being communicated (generating the P600). Finally, the representation constructed in the IFG feeds back to the pMTG via white matter tracts in the dorsal or ventral pathway, causing pre-activation of lexical features of possible upcoming words.

Putting the parts together, we can implement the Retrieval-Integration account in a cortical network, and walk through a typical processing cycle. Depending on whether the linguistic input is spoken or written, an incoming word reaches the lpMTG from respectively the auditory or the visual cortex. The lpMTG then acts as an epicenter for the retrieval of the lexical information associated with this word from the association cortices, where it is supposed to be stored in a distributed manner. The onset of this retrieval process corresponds to the onset of the N400 component, and ease with which semantic information can be retrieved corresponds to N400 amplitude. Via one of the candidate white matter tracts, the retrieved lexical information is then connected to the lIFG, where it will be integrated with a representation of the prior context into a representation of what is being communicated. The extent of work required to construct this updated representation corresponds to P600 amplitude. Finally, the representation constructed in the lIFG is fed back to the lpMTG via a white matter pathway resulting in the pre-activation of syntactic and semantic features of possible upcoming words. This Retrieval-Integration cycle is repeated as soon as a new word comes in. Figure 1 provides a schematic illustration of a typical Retrieval-Integration cycle.

Our functional-anatomical mapping of the Retrieval-Integration account predicts that each incoming word will first evoke activation in the lpMTG (retrieval) and subsequently in the lIFG (integration). A recent study using Event-Related Optical Signal (EROS) supports precisely this prediction (Tse et al., 2007); EROS responses to semantic and syntactic anomalies both showed sequences of increased activity in the lpTMG followed by activity in lIFG.

## 4. Discussion

We have proposed a new functional-anatomical mapping of the N400 component and the P600 component onto a minimal cortical network for language comprehension. In this mapping, the lpMTG (lexical retrieval) and lIFG (semantic integration) play a central role. Our mapping differs from earlier proposals by Friederici (2011) and Baggio and Hagoort (2011), which we have discussed in the introduction. We take the N400 component to index non-combinatory lexical retrieval processes mediated by the posterior temporal cortex, and the amplitude of the P600 to reflect the integration of the retrieved lexical information with a representation of prior context, which is hypothesized to take place in (or to be mediated by) the left inferior frontal regions. The account provided by Baggio and Hagoort (2011) is limited to the N400, which they assume to have generators both in the temporal lobe (reflecting the retrieval of lexical information), and in the frontal cortex (reflecting the construction of multi-word units). Our mapping also differs from the one proposed by Friederici (2011). Whereas Friederici (2011) assumes that the processes underlying the N400 are carried out in the temporal cortex (which is consistent with our view), she assumes these processes to involve combinatorial semantic processing (which contrasts with our non-combinatorial, retrieval view). Friederici (2011) is less specific about the P600, as she argues that “[a]t the linguistic level, the difficulty of integrating syntactic and semantic information and the need for reanalysis is reflected in a P600”, and that “[t]he difficulty of mapping linguistic information onto world knowledge also appears to elicit a P600 effect” (Friederici, 2011, p. 1383). Nonetheless, Friederici assumes these processes to be subserved by one particular area called the Temporo-Parietal Junction or TPJ (see Friederici, 2011, Figure 11) which clearly contrasts with our hypothesis that the P600 is generated in the lIFG.

### 4.1. The role of other cortical and sub-cortical areas

The mapping that we propose constitutes what we believe to be the core of the language comprehension system. This is not to say, however, that we believe the comprehension system to be limited to the lpMTG and the lIFG. We merely think that these areas are “epicenters” that form the *absolute core* of the comprehension network. Other cortical as well as sub-cortical areas are likely to also be important, but for most of them the role they play in language processing is still rather unclear. Moreover, even if we do have a clear idea of the processes sub-served by these areas, the coupling between electrophysiology and anatomy that we seek to arrive at requires us to also identify in which ERP component(s) these processes are reflected.

#### 4.1.1. Right hemisphere

One cortical area under discussion, for instance, is the Right Hemisphere. Friederici (2011) assumes that the Right Hemisphere is mainly host to prosodic processes, whereas Vigneau et al. (2011) argue against this, and conclude that at least the frontal part of the right hemisphere appears to be invoked whenever additional processing resources are required (e.g., when material in working memory needs to be manipulated). If Friederici's account is correct, activity in the right hemisphere might for instance contribute to late positivities that resemble the Closure Positive Shift (CPS; Steinhauer et al., 1999), which we argued is a likely member of the P600-family (Brouwer et al., 2012). On the other hand, if Vigneau et al. are right, processing in the right hemisphere might contribute to positivities that reflect substantial MRC revision. Further research into the role of the Right Hemisphere, especially the lpMTG and the lIFG homolog areas, is required to help us to decide between these hypotheses, and extend our mapping accordingly.

#### 4.1.2. Anterior temporal lobe (ATL)

Another cortical region that has recently received a vast amount of interest is the Anterior Temporal Lobe (ATL), the role of which is also much debated (see Bi et al., 2011; Tsapkini et al., 2011, for recent overviews). Bi et al. (2011) point out that there are at least two classes of hypotheses about the role of the ATL. On the one hand, the ATL is assumed to be a binding site for semantic properties (e.g., Rogers et al., 2004, 2006), supporting the view that it sub-serves the role of a “semantic hub” (Patterson et al., 2007)—however, see Binder and Desai (2011) for recent arguments against this interpretation. On the other hand, the ATL is assumed to be important for lexical retrieval (e.g., Damasio et al., 1996, 2004). On both of these accounts, activity in the ATL might contribute to the N400 component. Lau et al. (2008), however, argue that the ATL is more likely to support syntactic or thematic combinatorial processing. On this view, activity in the ATL rather contributes to the P600 component. Again, further research is required to help us to decide between these hypotheses and extend our present mapping as necessary.

#### 4.1.3. Angular Gyrus (AG)

Similarly, the Angular Gyrus (AG; BA39), a posterior part of the inferior parietal lobule, is also consistently implicated in language processing. In a recent review on AG function, for instance, Seghier (2013) discusses evidence for the AG's involvement in semantic processing and in word reading and comprehension, but also in number processing, attention and spatial recognition, memory retrieval, conflict resolution, and theory-of-mind/social cognition. In an attempt to unify these findings, Seghier argues that the AG is best defined as a cross-modal integrative hub that engages in event categorization, semantic access, retrieval of facts, and guidance of attention toward relevant information (see also Lau et al., 2008). If this view is correct, the question raises *if* and *how* the role of this hub in the parietal lobe is functionally distinct and/or complementary to the retrieval hub in the lpMTG. Interestingly, a potential answer to this question might be found in an hypothesis put forward by Binder and Desai (2011), who suggest that whereas the lpMTG hub might be more involved in the retrieval of conceptual representations of concrete entities, the AG hub may be geared more toward the retrieval of conceptual representations of *events*, which involve spatial and temporal interactions between entities. They exemplify this by means of the concept “birthday party”, the understanding of which requires the retrieval of a configuration of concrete entities, such as friends, cake, candles, and gifts, as well as the retrieval of a series of events that unfold in time and space, such as the lighting of the candles on the cake, and the opening of the gifts. If Binder and Desai are correct, we would predict activity in the AG to be reflected in the N400 component (on top of the activity in the lpMTG), reflecting retrieval of the conceptual event representations associated with (part of) the unfolding MRC in the lIFG.

#### 4.1.4. Sub-cortical areas

As for sub-cortical areas that have been implicated in language function, such as for instance the *basal ganglia* (e.g., Kotz et al., 2003), the *cerebellum* (e.g., Stowe et al., 2005), and the *thalamus* (e.g., Wahl et al., 2008), the same argument applies. We need to come up with clear hypotheses on the functional role of these areas in the comprehension system, before we can extend our functional-anatomic mapping to incorporate them. The core mapping that we have proposed is intended as a starting point for such further investigation into the functional role of these cortical and sub-cortical areas.

### 4.2. Parcellation of the lIFG and different P600s

Brouwer et al. (2012) hypothesized that the P600 component is a family of positivities, reflecting the word-by-word construction, reorganization, or updating of a mental representation of what is being communicated. Moreover, they argued that differences in amplitude, latency, duration, and scalp distribution of this component suggests that not every P600 is created alike (cf. Kaan and Swaab, 2003), and speculated that these electrophysiological properties may correlate with specific sub-processes that underlie the construction of a mental representation. In our functional-anatomic mapping, we put forward the hypothesis that the generation of every P600 is initiated in the lIFG, which raises the question of how differences in the electrophysiological properties of the P600 can arise. In our discussion of the functional role of the lIFG, we argued that it can be divided up into parcels, which may each have a distinct contribution to MRC construction. If different parcels of the lIFG are recruited for different sub-processes, this might explain (part of the) differences in electrophysiological properties of the P600 component. Another factor that might give rise to differences in electrophysiological properties, in particular scalp distribution, is when other nodes (cortical regions) in the neurocognitive network underlying language comprehension are invoked during integration. If, for instance, the right hemisphere homolog to the lIFG is indeed invoked in case of high processing demands (cf. Vigneau et al., 2011) or by the processing of prosodic information (cf. Friederici, 2011), the P600 component would then also reflect activity in the right hemisphere, which would affect its scalp distribution, but possibly also other properties like amplitude and duration. Interestingly, researchers have recently started categorizing differences in P600 properties (e.g., Van Petten and Luka, 2011). If our mapping is correct, this means that such a categorization of members of the P600 family might eventually turn out to have an anatomical basis: the different parcels of the lIFG. Moreover, this would mean that a finer-grained mapping of the different functions reflected in the P600 component to these different parcels might provide us with a detailed functional topology of the lIFG.

### 4.3. The dynamics of communication between the lIFG and lpMTG

The Retrieval-Integration account predicts that every incoming word modulates the amplitude of the N400 component (retrieval of lexical information), followed by a modulation of the P600 component (integration of the retrieved information). In terms of our mapping, this boils down to activity in the lpMTG (retrieval) followed by activity in the lIFG (integration). But the spatial separation of the epicenters for retrieval and integration predicts an additional source of activity reflecting the exchange of information between the lpMTG and the lIFG through the white matter tracts that connect them. However, as EEG measures post-synaptic potentials, rather than action potentials, we do not expect the activity in these white matter tracts to show up in the ERP signal. In addition, as hemodynamic responses may be restricted to gray matter, we also do not expect the information transfer between the lpMTG and lIFG to be visible in blood-flow based signals like the BOLD-response in fMRI (but see Mazerolle et al., 2010). This raises the question of how to study the dynamics of the information flow between the epicenters. One potential approach is to look at the pattern of oscillatory activity between the regions. Oscillatory activity reflects the synchronization of neuronal firing rates, and converging evidence suggests that such synchronization is related to the functional coupling of neural networks (engagement in cooperative processing), while desynchronization is related to their uncoupling (disengagement in cooperative processing; see Fries, 2005; Siegel et al., 2012). Studying the pattern of synchronization between the lpMTG and lIFG, using analysis techniques such as spectral coherence analysis (see Weiss and Mueller, 2003, for a review) or phase-locking statistics (Lachaux et al., 1999), may provide a window into the dynamics of the communication between these epicenters that can help to answer outstanding questions on the time-course of Retrieval-Integration cycles. For example, one could wonder whether the communication between the lpMTG and the lIFG is discrete or continuous. That is, it could be the case that information retrieved in lpMTG is immediately sent to the lIFG, independently of whether additional information is still being retrieved. The lIFG may then immediately attempt to integrate this, potentially incomplete information with a representation of what is being communicated. Under the Retrieval-Integration view, it is very likely that the lIFG is continuously active in creating a coherent representation of the situation that is currently attended. This activity will show an increase every time lexical information from a new incoming word becomes available. If this is correct, N400 and P600 components will overlap in time. As these components are generated independently, in different cortical regions or neurocognitive subnetworks, this means that they will then be summated in the resultant EEG signal. Hence, P600 amplitude may depend on preceding N400 amplitude, which may complicate the interpretation of late positivities following the N400 (for a discussion on this issue, see Hagoort, 2003). In future studies, we need to take this issue of potential overlap into account, and possibly change the way in which we analyze EEG data in order to properly disentangle retrieval and integration processes.

### 4.4. Predictions

To summarize, we will reiterate the above in terms of three concrete, falsifiable predictions that follow from our proposed functional-anatomical mapping of the Retrieval-Integration account, and that we believe are the most crucial for its validity:

1. **The P600 component is generated (in part) in the lIFG, and reflects MRC composition**. That is, we hypothesize that the generation of the P600 is initiated in the lIFG, but that the full-scale of activity reflected in P600 amplitude might include activity in other, recruited cortical regions as well. This is the most important prediction that follows from our mapping. Despite that there is as of yet no consensus on the neural generators of the P600 component, a number of fMRI studies have linked P600 amplitude to activity in the lIFG (see, van de Meerendonk et al., 2011, for a discussion). We did also find evidence for this prediction in our lab, as an unpublished fMRI version of the Hoeks et al. study on “Semantic Illusion” sentences (Hoeks et al., 2004) revealed a correlation between P600 amplitude and activity in the pars orbitalis (BA 47) of the lIFG.
2. **Different types of P600s (e.g., in terms of scalp distribution, onset, and/or amplitude) are generated (in part) in different parcels of the lIFG, and reflect different sub-processes of MRC composition**. That is, differences between P600s may arise because they are initiated in different parcels of the lIFG. However, whereas two distinct P600s may come about by activity in different lIFG parcels, they can also share activity in others, meaning that there may be overlap in the underlying generators of different P600s.
  Researchers have recently started to tease apart and categorize different types of P600s (see Van Petten and Luka, 2011, for instance). If our mapping holds, a categorization of functionally different P600s may have an anatomical basis in the functional topology of the lIFG. Moreover, it should be noted that if, for instance, Vigneau et al. (2011) are right in that the right hemisphere is invoked whenever processing demands are high, activity in the right hemisphere may also contribute to differences in P600s.
3. **Every incoming word produces activity in the lpMTG (lexical retrieval/~N400), followed by activity in the lIFG (integration/~P600)**. Preliminary EROS evidence for this prediction is provided by Tse et al. (2007), who found that both semantic and syntactic anomalies produced increased activity in the lpTMG followed by activity in lIFG. It might be that this pattern of activity occurs once for every incoming word (integration commences when retrieval is finished), or that activity alternates between the lpMTG and lIFG (integration commences as soon as a processable bit of meaning is retrieved). Albeit speculative, the aforementioned EROS study seems to suggest that the latter may be the case (see Tse et al., 2007, Table 1).

The first two predictions may be investigated using carefully aligned EEG and fMRI studies on stimuli that are known to *only* produce a P600-effect, such as syntactic violations, garden-path sentences, long-distance *wh*-dependencies, and thematic-role reversed “Semantic Illusion” sentences. If the first prediction is correct, we would expect all of these stimuli to produce increased activity in the lIFG, relative to an appropriate control. Moreover, if the second prediction holds, then these different stimuli may evoke activity in different sub-parts of the lIFG, producing characteristically different P600-effects in terms of onset, duration, and scalp-distribution, etc. The third prediction seems more challenging to test, but imaging techniques such as MEG or EROS might shed light on this issue.

## 5. Conclusions

We have proposed a minimal and parsimonious functional-anatomical mapping for language comprehension based on the Retrieval-Integration account (Brouwer et al., 2012). Our mapping entails that the processes of lexical retrieval, reflected in N400 amplitude, are mediated by the lpMTG. In the lIFG, lexical information retrieved in lpMTG is integrated with a representation of its prior context into a representation of what is being communicated. These integrative processes are reflected in P600 amplitude. The representation constructed in the lIFG subsequently provides the lpMTG with retrieval cues for the next word, upon which the Retrieval-Integration cycle repeats itself.

We argued that our mapping forms the core of the comprehension system, and we want to stress that we do not believe the comprehension system to be limited to this minimal, core network. In future research, our mapping—which is based on the “epicenters” of the language network—may be extended to incorporate other cortical areas, such as regions in the right hemisphere (e.g., the homologs to the lIFG and lpMTG), the ATL, and the AG, which have also been implicated in language comprehension, but the precise function of which is still unclear. Critically, such an extension will require us to identify the function of these regions, and ascertain what kinds of ERP components (e.g., ELAN/LAN/Sustained Negativities) result from the activation of these areas. The present mapping may serve as a starting point for such an endeavor.

The proposed correlation between activity in the lIFG and P600 amplitude also paves way for further, more fine-grained investigations into the relation between electrophysiology and brain anatomy. Earlier (Brouwer et al., 2012), it has been hypothesized that the P600 is a family of late positivities, of which different members may reflect different processes involved MRC construction. In the present paper, we have suggested that these differences in electrophysiological properties of the P600, might arise because different instances of the P600 are generated in the different parcels of lIFG. This means that a categorization of different P600-effects (Van Petten and Luka, 2011), and a mapping of this categorization to the different parcels of the lIFG, might provide a means of uncovering a fine-grained functional topology of this area.

### Conflict of interest statement

The authors declare that the research was conducted in the absence of any commercial or financial relationships that could be construed as a potential conflict of interest.
